# Supplementary material for: Photonic-crystal exciton-polaritons in monolayer semiconductors
Source: Nat Commun. 2018 Feb 19;9:713. doi: 10.1038/s41467-018-03188-x (PMC5818602; doi:10.1038/s41467-018-03188-x)
Supplement: Supplementary file 1 — Supplementary Information [file 41467_2018_3188_MOESM1_ESM.pdf]

Photonic-Crystal Exciton-Polaritons in Monolayer Semiconductors

Long Zhang[1], Rahul Gogna[2], William Burg[3], Emanuel Tutuc[3] and Hui Deng[1,2*]

[1] *Physics Department, University of Michigan,*

450 Church Street, Ann Arbor, MI 48109-2122, USA

[2] *Applied Physics Department, University of Michigan,*

450 Church Street, Ann Arbor, MI 48109-1040, USA and

[3] *Microelectronics Research Center, Department of Electrical and Computer Engineering,*

The University of Texas at Austin, Austin, Texas 78758, United States

[*] dengh@umich.edu

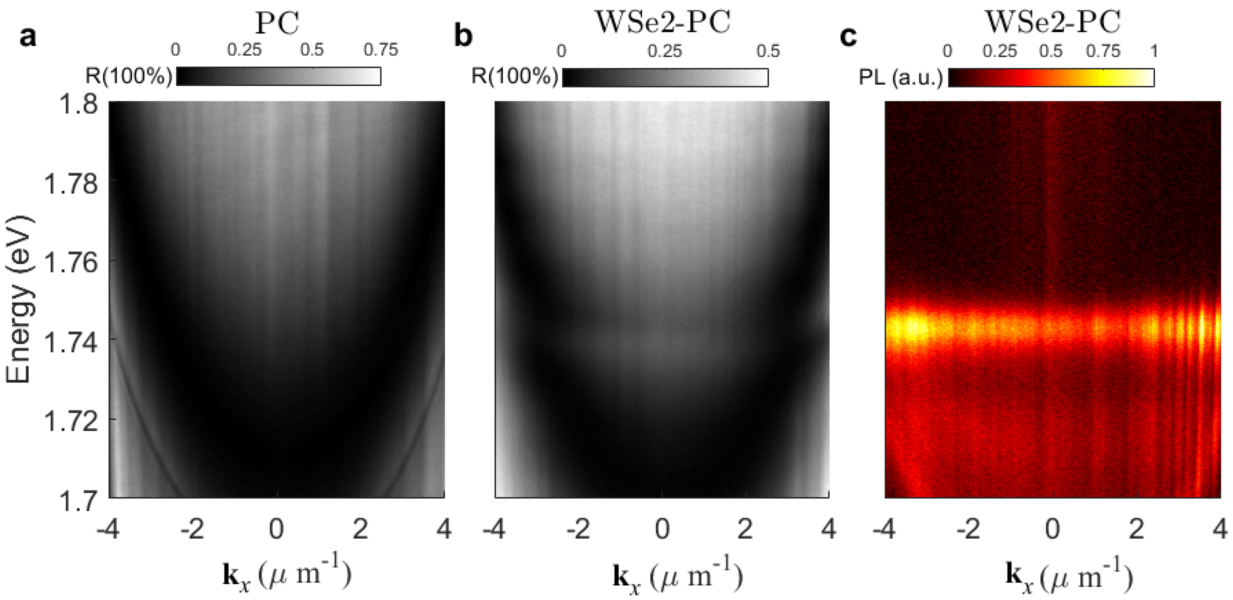

Supplementary Figure 1: The TM-polarized modes of the WSe_2-PC device measured by angle-resolved reflectance (a-b) and PL (c) at 10 K. a, Reflectance of the bare PC, showing the absence of any PC modes near the WSe_2 exciton resonance. b, Reflectance of the WSe_2-PC device, showing only weak absorption by the uncoupled, dispersionless TM exciton at $E_{\mathrm{exc}} \sim 1.742$ eV. c, PL from the WSe_2-PC device, showing the dispersionless TM exciton emission. Due to the absence TM-polarized PC modes near exciton resonance, TM excitons remain in the weak coupling regime, which provides direct reference for the energies of the un-coupled exciton mode.

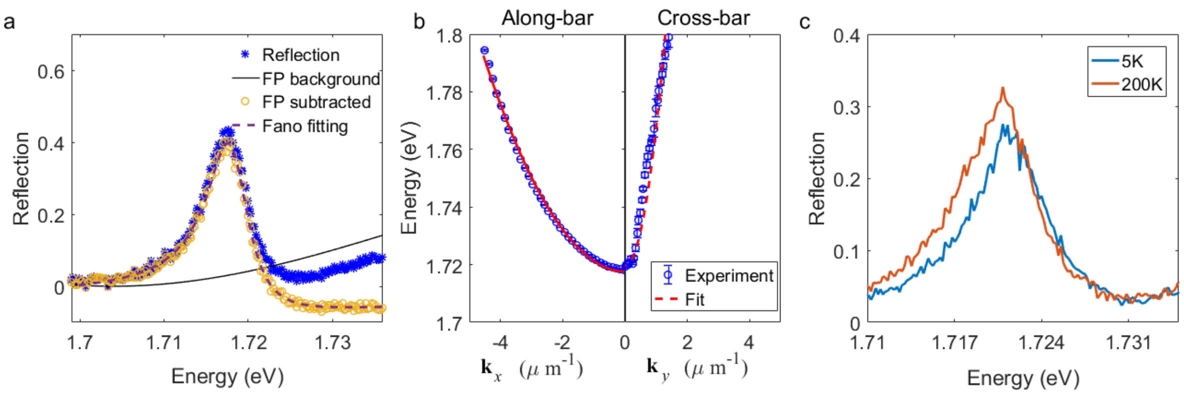

Supplementary Figure 2: Characterization of the TE-polarized PC-modes: linewidth, dispersion, and temperature dependence. a, The measured total reflectance spectrum from the WSe_2-PC device at zero degrees for the TE-polarization (blue stars). It can be decomposed to the FP-background, which we calculate by the transfer matrix method (black line), and reflectance of the PC-mode (yellow circles) obtained by subtracting the FP background. The reflectance of the PC-mode features a Fano line shape, which we fit using equation (4) in the main text and obtain the cavity linewidth $\gamma_{\text{cav}} = 3 \pm 0.5$ meV. The asymmetry parameter is $q = 4.6 \pm 0.17$, which is much larger than 1 and indicates that the line shape is close to Lorentzian. b, Anisotropic dispersions of the PC-mode obtained from angle-resolved reflectance measurement of the bare PC. Due to the inplane anisotropy of grating structure, the dispersion of the PC-modes are highly anisotropic. The left panel shows the dispersion in the along-bar direction, or x-direction, which has a smaller curvature and is well-described as parabolic in the angle range we measured. The blue circles are the measured resonance energies obtained by fitting spectra in Fig.2 using Fano lineshape, and the red line is fitting by $E = \alpha(\mathbf{k}_x)^2 + E_0$, with fitted parameters E_0=1.7189±0.1 $\times$ 10^{-3} eV and α=3.6$\times$$10^{-3}$ $\pm 0.1 \times 10^{-3}$, in which k is in the unite of μm^{-1}. The right panel shows the dispersion in the cross-bar direction, or y-direction, which has a much larger curvature. The blue circles are the measured resonance energies obtained by fitting spectra in Fig.2 using Fano lineshape, and red line is fitting by $E = \beta(\mathbf{k}_y)^2 + E_0$, with fitted parameters $\beta = 0.047 \pm 0.003$. All the errorbars are extracted by least square data fitting. c, Reflectance spectra of the bare PC at $\theta_x = 5°$ measured at 5 K and 200 K. The two spectra show only a slight shift of < 1 meV.

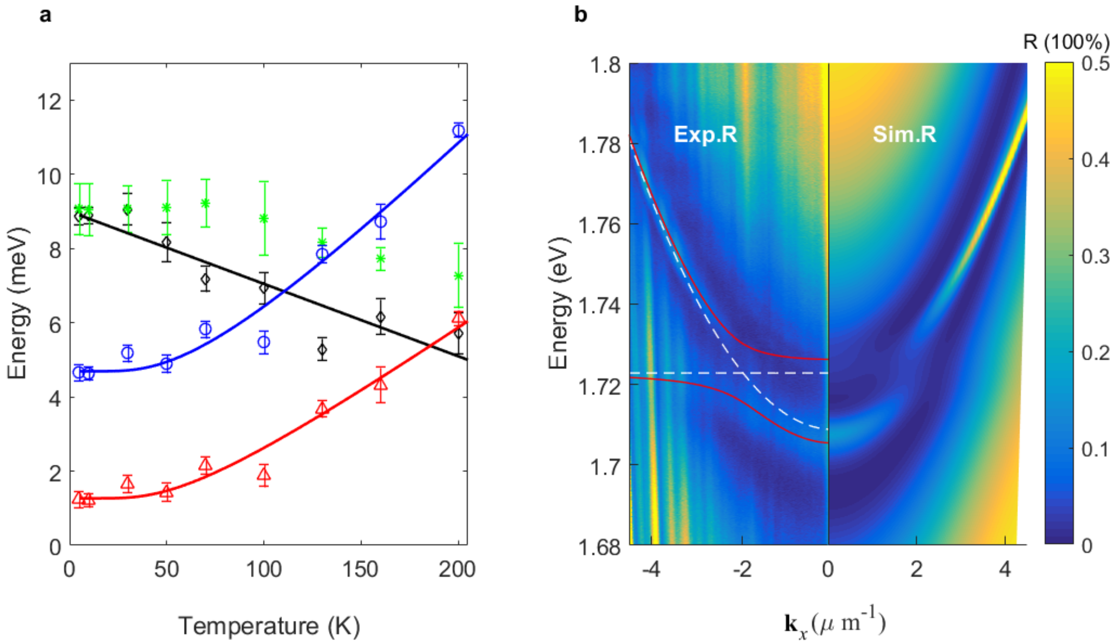

Supplementary Figure 3: Reflectance spectra in the weak-coupling regime. a, Comparison of the temperature dependence of the coupling strength g (black diamonds) obtained from fitting of the dispersion measured via PL, g_R (green stars) obtained from fitting of the dispersion measured via reflection, $\sqrt{(\gamma_{exc}^2 + \gamma_{\rm cav}^2)/2}$ (blue circles) and $(\gamma_{\rm exc} - \gamma_{\rm cav})/2$ (red triangles). It shows that system transitions to the weak coupling regime above 110 K, while g_R is consistently higher than g and would indicate strong coupling up to about 140 K. All the errorbars are extracted by least square data fitting. b, Angle-resolved reflection spectra of WSe_2-PC at 130 K. We fit this measured dispersion with the coupled oscillator model (red lines). The white dashed lines are the dispersion of exciton and PC modes. Anti-crossing is still evident in the reflection spectra, despite that the system is already in the weak-coupling regime where $g < \sqrt{(\gamma_{\rm exc}^2 + \gamma_{\rm cav}^2)/2}$. The fitted $g_R \sim 8.2 \pm 0.4$ meV is well above $g \sim 5.3 \pm 0.3$ meV, the latter is obtained from PL spectra and is a more accurately value for the coupling strength. This example illustrates that reflection spectra can give misleading conclusion on strong-coupling. Likewise, transmission spectra would produce an even larger mode splitting than that of reflection spectra when in the weak coupling regime [1].

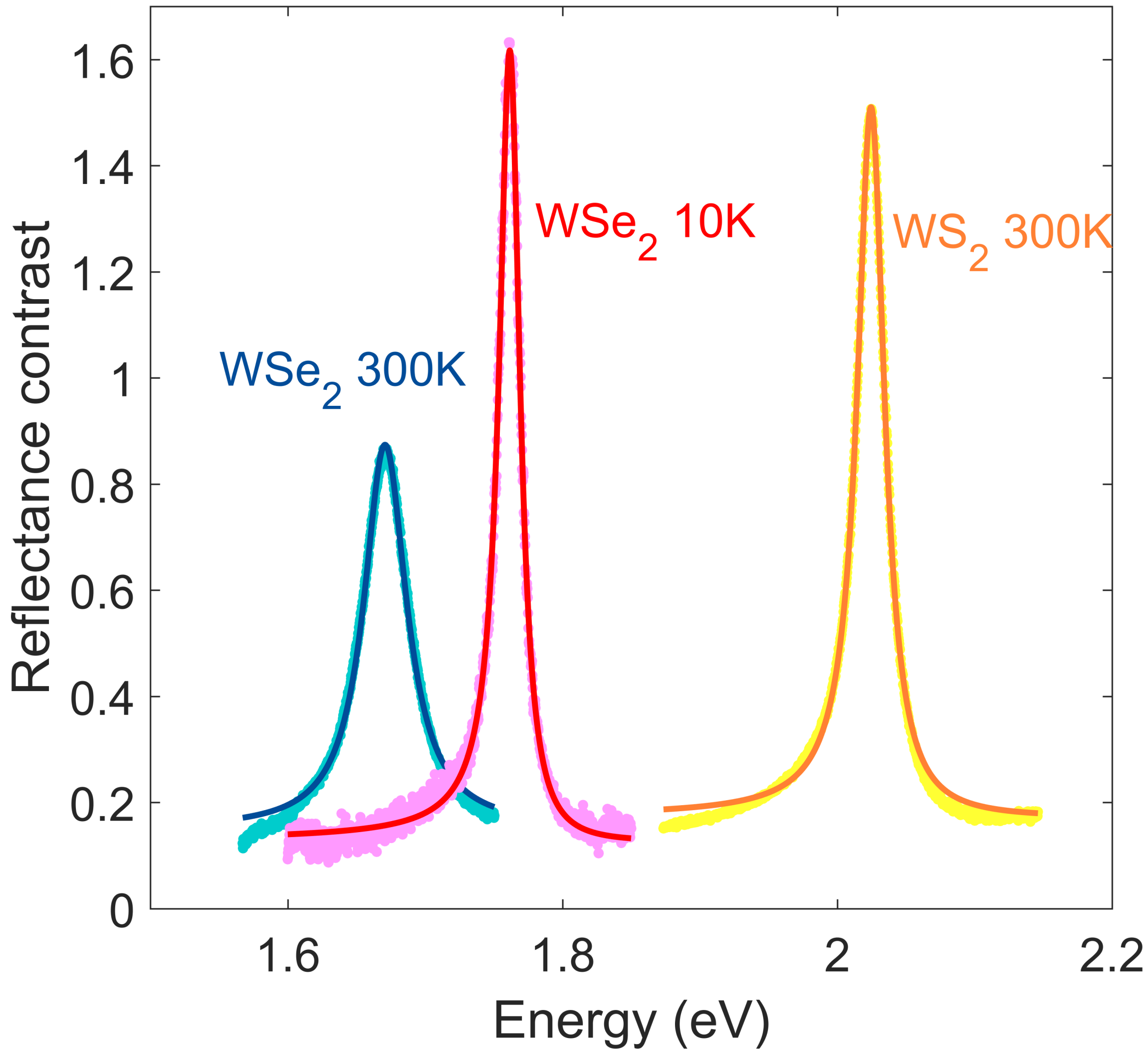

Supplementary Figure 4: Comparison of oscillation strength and linewidth of WSe_2 and WS_2 monolayers. Reflection contrast spectra of a WSe_2 monolayer measured at 10 K and 300 K, and a WS_2 monolayer at 300 K (dots). The reflection contrast is obtained as $R_{contrast} = R_{TMD}/R_{sub} - 1$, where R_{TMD} is reflectance from the monolayer TMDs on a glass slide substrate, and R_{sub} is the reflectance from the glass slide alone. The solid lines are fits using transfer matrix calculation. In the case of monolayer sample, absorption is approximately linear with the $R_{contrast}$ [2, 3]. Thus, the WS_2 monolayer has a stronger absorption and narrower linewidth compred to those of WSe_2 at 300 K, in agreement with the reported results [4], which suggests WS_2 to be more suitable for room temperature polaritons.

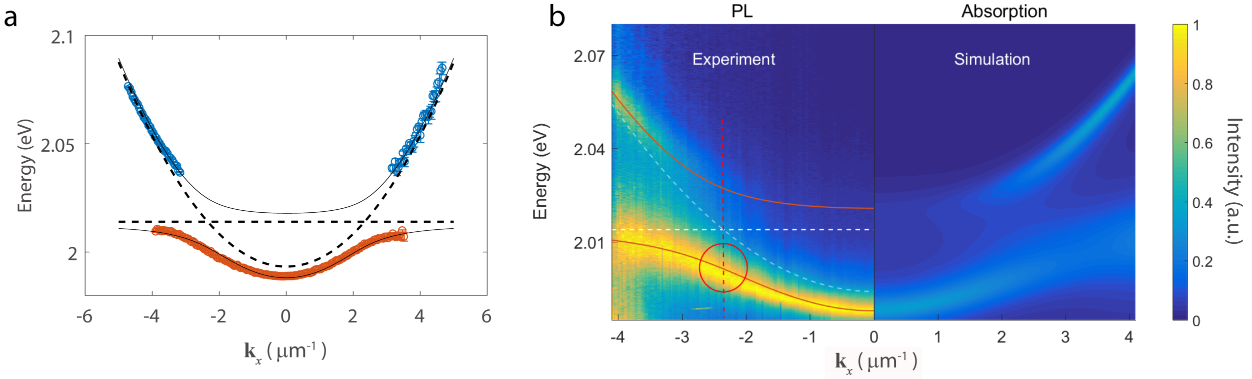

Supplementary Figure 5: Dispersion of the WS_2-PC integrated device. (a) The k-resolved PL spectra of WS_2-PC integrated device with the intensity normalized for each wavenumber (left panel), compared with the simulated absorption spectra (right panel). (b) The polariton dispersions. The symbols are the measured resonance energies obtained by Gaussian fits of the spectra at each wavevector in (a), and The errorbars are extracted by least square data fitting. The solid lines are fits to the measured dispersion by the polariton dispersion relations in Eq. 1 of the main text. The dashed lines are the un-coupled exciton and photon dispersions from the fits. The exciton energy E_{exc}, exciton photon coupling strength g, and the bare cavity energy at zero wavevector $E(\mathbf{k}_x=0,\mathbf{k}_y=0)$ are the fitting parameters. The raw data shown in Figure 4a (left panel) in the main text show both the dispersion and the intensity distribution among different k-modes, as a result the modes with weaker intensity are less visible in the figure. Here we normalize the PL intensity for each wave-vector to enhance the visibility at larger wavevectors, as shown in a. We marked the zero detuning by a red dashed line; in this case, the inflection point also falls in the region around zero-detuning, as marked by the red circle. (c)-(e), three example spectra at different wavevectors with Gaussian fits

Supplementary Table 1: Parameters to calculate the Fano line shape in Figure 5a,b

parameters	WSe_2 device			WS_2 device		
	PC	LP	UP	PC	LP	UP
$\|R_F\|$	0.016	0.012	0.008	0.11	0.04	0.10
ω_0	1.764	1.739	1.761	2.048	1.972	2.048
γ_0 (meV)	3.1	12	5	5.2	7.2	5.7
q	5	3.5	4.1	0.93	0.83	0.18
I_b	-0.0162	-0.072		0.027	0.0073	
thickness of Si_3N_4 (nm)	113			78		
thickness of SiO_2 (nm)	1475			2000		

SUPPLEMENTARY REFERENCES

[1] Savona, V., Andreani, L. C., Schwendimann, P. & Quattropani, A. Quantum well excitons in semiconductor microcavities: Unified treatment of weak and strong coupling regimes. *Solid State Communications* **93**, 733–739 (1995).

[2] Mak, K. F. *et al.* Measurement of the Optical Conductivity of Graphene. *Physical Review Letters* **101** (2008).

[3] Mak, K. F., Lee, C., Hone, J., Shan, J. & Heinz, T. F. Atomically Thin MoS_2 : A New Direct-Gap Semiconductor. *Physical Review Letters* **105** (2010).

[4] Li, Y. *et al.* Measurement of the optical dielectric function of monolayer transition-metal dichalcogenides: MoS_2 , $MoSe_2$, WS_2 , and WSe_2. *Physical Review B* **90** (2014).
